# Compétences des couples en matière de planification familiale en post-partum immédiat dans le District de Santé de Biyem-Assi, Cameroun

**DOI:** 10.11604/pamj.2019.32.172.15050

**Published:** 2019-04-09

**Authors:** Nadia Maguiassué Téfouet, Marius Zambou Vouking, Marie-José Essi

**Affiliations:** 1 Evidence pour les Systèmes de Développement Humain Durable en Afrique, Nouvelle Route Bastos, Yaoundé, Cameroun; 2 Ecole des Sciences de la Santé de l’Université Catholique d’Afrique Centrale, Messa, Yaoundé, Cameroun; 3 Centre pour le Développement des Bonnes Pratiques en Santé, Hôpital Central de Yaoundé, Henri-Dunant Avenue, Messa, Yaoundé, Cameroun; 4 Université de Yaoundé I, Département de Santé Publique, Yaoundé, Cameroun

**Keywords:** Compétences, couples, post-partum immédiat, planification familiale en post-partum immédiat, Contraceptive practices, immediate postpartum period, family planning in the immediate postpartum period

## Abstract

**Introduction:**

La planification familiale en post-partum immédiat reste encore peu connue et peu pratiquée par la plupart des couples au Cameroun. Pourtant, juste après un accouchement, nombreux sont ceux qui désirent différer la naissance de leur prochain enfant. Cette étude avait pour objectif de déterminer le niveau de compétences et le besoin éducationnel des couples en matière de planification familiale en post-partum immédiat dans le District de Santé de Biyem-Assi.

**Méthodes:**

Il s'agissait de mener une enquête CAP (connaissances, attitudes et pratiques), dans le District de Santé de Biyem-Assi. La collecte des données s'est faite à l'aide de deux questionnaires de 40 questions chacun, rédigés en français, l'un adressé aux femmes en couple et en post-partum et l'autre aux hommes en couple et ayant au moins un enfant. Les données recueillies ont été saisies dans le logiciel CSPro version 6.2, puis analysées à l'aide du logiciel SPSS version 20.0.

**Résultats:**

Un total de 300 individus a été interrogé avec un sex-ratio de 1. Plus de la moitié (56,7%) avait une connaissance approximative de la planification familiale en post-partum immédiat. Pour 36% des répondants, le post-partum immédiat n'était pas un moment approprié pour l'utilisation d'une méthode contraceptive moderne, l'interaction entre contraceptifs et lait maternel (65,4%) et l'infertilité de la femme (26,3%) étaient les principales raisons évoquées. Les pratiques contraceptives en post-partum immédiat de 60,5% des couples vivant dans le District de Santé de Biyem-Assi étaient inadéquates. Par conséquent, la plupart des enquêtés avaient un niveau de compétences insuffisant (32,6%) et faible (23,3%) en matière de planification familiale en post-partum immédiat.

**Conclusion:**

Certains préjugés et de fausses idées persistent dans la population du District de Santé de Biyem-Assi en ce qui concerne les méthodes contraceptives modernes, et constituent un frein à la pratique contraceptive en général et en post-partum immédiat en particulier. Des efforts de sensibilisation et d'éducation des couples pour l'amélioration de leurs compétences en matière de contraception en post-partum immédiat s'avèrent nécessaires.

## Abstract (en)

**Introduction:**

Postpartum family planning is still little known and little practised by most couples in Cameroon. However, just after childbirth, many couples wish to postpone their second birth. This study aims to determine the level of competence and the education need of couples in immediate postpartum family planning in the Biyem-Assi health district, Cameroon.

**Methods:**

We performed a KAP survey (knowledge, attitudes and practices) in the Biyem-Assi health district. Data were collected using two questionnaires with 40 questions, written in french, one addressed to women in couples and during the post-partum period and the other addressed to men in couples and having at least one child. Collected data were entered in the software CSPro version 6.2 and then analyzed using the software SPSS version 20.0.

**Results:**

A total of 300 subjects with a sex ratio of 1 were surveyed. More than half (56.7%) had an approximate knowledge of family planning in the immediate postpartum period. Thirty six percent of the respondents believed that the immediate postpartum period was not an appropriate time to use a modern method of contraception. The interaction between contraceptives and breast milk (65.4%) and female infertility (26.3%) were the main reasons reported. Immediate postpartum contraception of 60.5% of couples living in the Biyem-Assi health district was inadequate. Therefore, the level of competence in the immediate postpartum family planning was insufficient (32.6%) and low (23.3%) in most of the respondents.

**Conclusion:**

Some prejudices and misconceptions about modern contraceptive methods persist in the Biyem-Assi health district population and these are an obstacle to contraceptive practice in general and during the immediate postpartum period in particular. Awareness and education efforts of couples to improve their competence in contraception during the immediate postpartum period are necessary.

## Introduction

La planification familiale (PF) est un pilier majeur des soins de santé reproductive qui peut être dispensée avant la grossesse, immédiatement après l'accouchement et pendant la première année suivant l'accouchement [1]. Elle est importante tout au long de la vie génésique d'un couple ou d'un individu [1], et constitue un moyen d'atteindre le nombre d'enfants souhaité et de déterminer l'espacement entre les naissances [2]. La planification familiale du post-partum (PFPP) quant à elle revêt un intérêt particulier pour la prévention des grossesses rapprochées et non désirées pendant les 12 premiers mois qui suivent un accouchement [1]. Dans le cadre des programmes typiques de santé maternelle et néonatale, la période post-partum est définie comme étant les six semaines qui suivent une naissance, mais dans le contexte de la PFPP, cette période fait allusion à la première année après une naissance [3]. Cette étude se rapportant à la PFPP, nous avons considéré les six semaines qui suivent une naissance comme période de post-partum immédiat (PPI) dans les couples.

En 2015, les estimations globales de l'Organisation Mondiale de la Santé (OMS) ont révélé que 303 000 femmes dans le monde, âgées de 15 à 49 ans, meurent chaque année, suite aux complications de la grossesse, de l'accouchement et des suites de couches [4]. Près de 99% de ces décès surviennent dans les pays en développement, l'Afrique Subsaharienne représentant à elle seule 66% [4, 5]. Le taux mondial de mortalité infantile, estimé à 43 décès pour 1 000 naissances vivantes en 2015 est tout aussi alarmant, l'Afrique Subsaharienne possédant le taux le plus élevé du monde avec un enfant sur douze qui meurt avant son cinquième anniversaire [6]. Au Cameroun, ces taux sont respectivement établis à 782 décès maternels pour 100 000 naissances vivantes selon l'Enquête Démographique et de Santé de 2011 (EDS-MICS 4) [7] et 103 décès d'enfants de moins de 05 ans pour 1 000 naissances vivantes selon la dernière Enquête par grappe à indicateurs multiples (MICS 5) [8]. Une grande proportion de ces décès (70%) est due aux facteurs liés à la grossesse, plus particulièrement aux grossesses non désirées ou à haut risque qu'on résume généralement par les quatre « trop » classiques: trop tôt, trop nombreuses, trop tardives et trop rapprochées [9]. Des études ont montré que si tous les couples des pays en développement espaçaient leurs grossesses d'au moins 18 mois, les décès maternels dans le monde diminueraient d'environ 32% et les décès infantiles de près de 10% [10, 11]. En effet, les grossesses qui surviennent moins de 18 mois après la naissance d'un frère ou d'une sœur présentent des risques significativement plus élevés de morbidité et mortalité maternelle et infantile, et accroissent les risques de complications néonatales telles que la prématurité, un faible poids de naissance et une petite taille pour l'âge gestationnel [11-13].

Juste après un accouchement, bien de couples souhaitent différer la naissance de leur prochain enfant d'au moins 2 à 3 ans [14], mais les femmes en post-partum sont celles ayant le plus grand besoin non satisfait en planification familiale (PF) [1]. De nombreuses méthodes contraceptives modernes (MCM) peuvent pourtant être utilisées par les couples à n'importe quel moment après un accouchement et même en post-partum immédiat (PPI) [1]. Nombreux pays en développement à l'instar du Burkina Faso, de la République Démocratique du Congo, de la Guinée et de la Zambie ont déjà pris l'initiative d'intégrer les services de PF dans le continuum des soins postnataux immédiats par insertion du dispositif intra-utérin chez les femmes consentantes [15]. Mais au Cameroun, la documentation sur la planification familiale du post-partum immédiat (PFPPI) est encore très faible aujourd'hui. Un projet pilote «E2A» (Evidence to Action) dirigé par Pathfinder International, consistant à mettre en place des services de PFPPI, a été mis en œuvre dans six hôpitaux de la ville de Yaoundé dont l'Hôpital de District de Biyem-Assi en faisait partie. Les résultats sont satisfaisants et prometteurs, mais la population semble encore peu informée et les pratiques contraceptives des couples en PPI, toujours inadaptées. Leurs compétences en matière de PFPPI semblent encore très limitées et inadéquates. Selon l'OMS, les compétences sont des *« Connaissances, et aptitudes et attitudes communicationnelles, psychomotrices et décisionnelles suffisantes pour exécuter des actions et des tâches spécifiques avec un niveau de maîtrise défini »* [16]. Pour posséder ces compétences en matière de santé, il faudrait avoir un bon niveau de connaissances et posséder des attitudes justes qui permettent de prendre des mesures requises pour améliorer sa santé et celle de la population [17]. Aussi, la présente étude se proposait-elle de décrire les pratiques contraceptives des couples en PPI afin de déterminer leur niveau de compétences et leur besoin éducationnel en matière de PFPPI, notamment dans le District de Santé de Biyem-Assi.

## Méthodes

**Type d'étude:** nous avons mené une étude portant sur les connaissances, attitudes et pratiques (étude CAP), quantitative et à visée descriptive.

**Description du milieu d'étude:** le district de Santé (DS) de Biyem-Assi est l'un des six districts que compte la ville de Yaoundé, bien qu'il y existe des poches urbano-rurales surtout dans les zones d'accès difficile. Il s'étend sur une superficie de 22km^2^ et couvre l'arrondissement de Yaoundé VI^ème^ d'une population cosmopolite estimée à environ 358 335 en 2016 repartie dans onze aires de santé [18]. Il compte 93 formations sanitaires parmi lesquelles 09 publiques, 04 privées confessionnelles et 80 privées laïques dont la plupart fournissent des services de planification familiale [18].

**Sélection des sujets:** les hommes et femmes en couple vivant dans la zone géographique du DS de Biyem-Assi depuis au moins un an, ou venus consulter dans l'une des formations sanitaires dudit DS et ayant consenti à participer à l'enquête ont été inclus. Etant donné que l'étude se rapportait à la pratique contraceptive dans une période particulière de la vie d'un couple (le Post-Partum Immédiat), seuls les hommes ayant au moins un enfant et les femmes en post-partum et qui avaient connaissance de l'existence d'au moins une méthode contraceptive moderne (MCM) ont été interrogés. Le nombre total de personnes à interroger a été déterminé en fonction de la prévalence contraceptive chez les femmes de 15-49 ans au Cameroun, 24% selon EDS IV [7], soit un échantillon de 300 individus. Par la suite l'échantillon a été réparti en fonction du sexe et de l'âge afin d'avoir un échantillon raisonné.

**Collecte des données:** les données ont été collectées à l'aide de deux questionnaires structurés, de 40 questions chacun (l'un pour les hommes et l'autre pour les femmes) préalablement pré-testés et validés. Les questions portaient principalement sur: les connaissances de l'usage des MCM immédiatement après un accouchement, les sources et le moment de réception de l'information, les attitudes vis-à-vis de la sexualité et de la PF en PPI, le moment de reprise de la sexualité en post-partum, les conseils reçus à la sortie de la maternité et l'utilisation ou non d'une MCM en PPI.

**Considérations éthiques:** cette étude a obtenu l'autorisation de l'Ecole des Sciences de la Santé de l'Université Catholique d'Afrique Centrale et des autorités sanitaires du DS de Biyem-Assi pour être menée. Avant le début de la collecte des données, une clairance éthique du Comité Ethique Institutionnel de la Recherche pour la Santé Humaine a également été obtenue. Un consentement éclairé verbal était requis pour chaque participant avant l'administration du questionnaire. Pendant le remplissage de la fiche d'enquête, les sujets ont été identifiés à l'aide d'un code unique à chacun et aucune donnée individuelle (nom, prénom, numéro de téléphone) n'a été enregistrée. Les données collectées ont été conservées en lieu sécurisé et n'étaient accessibles qu'à un noyau de personnes clés autorisées.

**Analyse des données:** les données recueillies ont été saisies dans le logiciel CSPro version 6.2, puis analysées à l'aide du logiciel SPSS version 20.0. Les résultats ont été quantifiés et interprétés à l'aide de la grille d'analyse définie par Essi *et al.* [19], ainsi qu'il suit: le niveau de connaissance des couples en matière de PFPPI a été restitué en 04: mauvais (moins de 25% de bonnes réponses), insuffisant (moins de 50% de bonnes réponses), moyen (moins de 70% de bonnes réponses) et bon (plus de 70% de bonnes réponses); l'analyse des attitudes des couples vis-à-vis de la PFPPI a été faite en 04 catégories: juste, approximative, erronée et néfaste; et l'analyse des pratiques contraceptives a été établie en 03 niveaux: néfastes, inadéquates et adéquates. Les compétences des couples ont été évaluées par association de leurs connaissances, attitudes et pratiques contraceptives, et restituées en 04 niveaux: mauvaises (moins de 25% de bonnes réponses), insuffisantes (moins de 50% de bonnes réponses), moyennes (moins de 70% de bonnes réponses) et bonnes (plus de 70% de bonnes réponses).

## Résultats

Un total de 300 individus a été interrogé, avec un sex-ratio de 1. L'âge moyen des hommes était de 36 ± 2,5 ans et celui des femmes de 30 ± 2 ans. Les populations hommes et femmes ont été équitablement réparties entre les jeunes et les adultes. Le nombre moyen de grossesses par femme était de 3 pour une variation comprise entre 1 et 8 grossesses. Près de 31% de ces femmes ont affirmé que leur dernière grossesse n'était pas planifiée. Chez les hommes, le nombre moyen d'enfant par homme était de 3,5 pour une variation comprise entre 1 et 14 enfants et le nombre de partenaires sexuelles était compris entre 1 et 7 pour une moyenne de 2 partenaires par homme.

**Figure 1:**
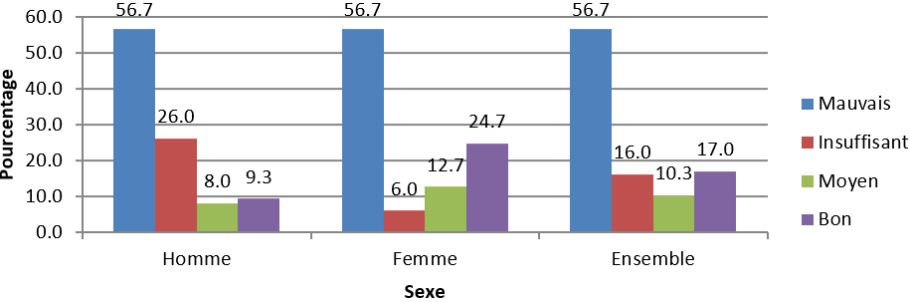
Niveau des connaissances en matière de PFPPI en fonction du sexe

**Connaissances en matière de PFPPI:** la connaissance de l'utilisation des MCM en PPI était faible dans la population étudiée. Seuls 37,6% de la population totale, soit 43,3% de femmes contre 32% d'hommes, avaient déjà été informées qu'au moins une de ces méthodes pouvait être utilisée en PPI (Tableau 1). Le préservatif (100%) et les pilules orales (44%) étaient les méthodes les plus citées. Plus de la moitié (53,4%) avait reçu l'information d'un personnel de santé, mais 26,3% étaient peu satisfaits de l'information reçue. Le retour des couches était assimilé au retour à la fécondabilité chez une femme en post-partum par 46% des enquêtés (Tableau 1). L'analyse du niveau des connaissances des enquêtés en matière de PFPPI, selon la grille de Essi *et al.* [19], montre que la proportion des personnes ayant un bon niveau des connaissances était très faible (17%) avec celui des femmes 2,6 fois plus élevé que celui des hommes (Figure 1).

**Tableau 1:** Connaissances des couples en matière de PFPPI

Variables	Hommes [% (n)]	Femmes [% (n)]	Total [% (n)]
**Connaissances des couples en matière de PFPPI**			
**Possibilité d’utilisation d’une MCM en PPI**			
Oui	32,0 (48)	43,3 (65)	37,7 (113)
Non	68,0 (102)	56,7 (85)	62,4 (51)
**Méthodes utilisables en PPI**			
Préservatif	100,0 (48)	100,0 (65)	100,0 (113)
Méthodes naturelles	41,7 (20)	47,7 (31)	45,1 (51)
Pilules orales	25,0 (12)	49,2 (32)	38,9 (44)
Pilules injectables	25,0 (12)	38,5 (25)	31,7 (37)
DIU	16,7 (8)	33,8 (22)	26,5 (30)
**Réception de l’information**			
A un moment quelconque	20,7 (31)	20,0 (30)	20,3 (61)
Pendant la grossesse	7,3 (11)	12,7 (19)	10,0 (30)
Un mois après l'accouchement	0,0 (0)	5,3 (8)	2,7 (8)
Dès la naissance de l'enfant	3,3 (5)	0,0 (0)	1,7 (5)
**Sources d’information**			
Personnel de santé	31,3 (15)	75,4 (49)	53,4 (64)
Ecole	27,1 (13)	32,3 (21)	29,7 (34)
Média	37,5 (18)	20,0 (13)	28,7 (31)
Famille	18,8 (9)	18,4 (12)	18,6 (21)
**Fécondabilité en PP**			
Retour des couches	47,3 (71)	44,7 (67)	46,0 (138)
25^ième^ jour	16,7 (25)	30,0 (45)	23,3 (70)
Ne sais pas	24,0 (36)	14,0 (21)	19,0 (57)
Premier jour	10,0 (15)	10,7 (16)	10,3 (31)
Aucun risque	2,0 (3)	0,7 (1)	1,3 (4)

**Figure 2:**
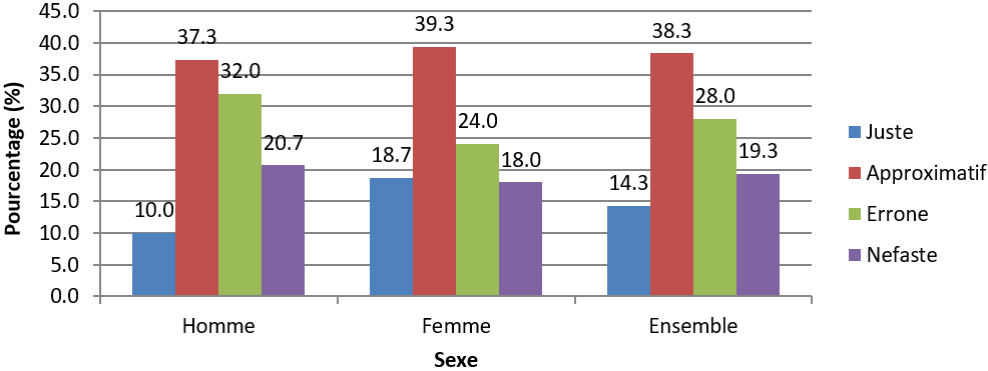
Attitudes vis-à-vis de la PFPPI en fonction du sexe

**Attitudes vis-à-vis de la sexualité et de la PF en PPI:** une proportion importante de 43,1% des enquêtés reprend très tôt avec les rapports sexuels en post-partum dans leur couple, mais 36% de ces derniers pensaient que le PPI n'était pas une période appropriée pour l'adoption d'une MCM. L'interaction entre les contraceptifs et lait maternel (65,4%) et l'infertilité de la femme au cours de cette période (26,3%) étaient les principales raisons évoquées (Tableau 2). La possibilité d'une utilisation future était non envisageable pour plus de la moitié de l'échantillon (55%), principalement par peur des effets des contraceptifs sur la santé de la femme et de l'enfant (70,5%), notamment en ce qui concerne les méthodes hormonales et mécaniques (Tableau 2). L'analyse des attitudes globales selon la grille de Essi *et al.* [19] a montré que 38,3% (37,3% des hommes et 39,3% des femmes) avaient des attitudes approximatives vis-à-vis de la PFPPI (Figure 2). En effet, ils pouvaient avoir des rapports sexuels dans les jours ou semaines qui suivent un accouchement, mais pensaient qu'il n'était pas nécessaire d'utiliser une MCM à cette période.

**Tableau 2:** Attitudes des couples vis-à-vis de la sexualité et de la PF en PPI

Variables	Hommes [% (n)]	Femmes [% (n)]	Total [% (n)]
**Attitudes des couples vis-à-vis de la sexualité en PPI**			
Favorable			
Intégrité du couple	46,0 (29)	34,0 (20)	40,0 (49)
Satisfaction du partenaire	28,6 (18)	49,1 (29)	38,8 (47)
Satisfaction du désir personnel	25,4 (16)	16,9 (10)	21,2 (26)
Défavorable			
Interdit culturel (priorité à l'enfant)	41,4 (36)	30,8 (28)	36,1 (64)
Fatigue/fragilité de la femme	13,8 (12)	52,7 (48)	33,2 (60)
Impact sur le lait maternel	17,2 (15)	16,5 (15)	16,8 (30)
Recours à d'autres partenaires	27,6 (24)		
**Attitudes des couples vis-à-vis de la PFPPI**			
**PPI approprié pour adopter une MCM**			
Oui	49,3 (74)	59,3 (89)	54,3 (163)
Non	38,0 (57)	34,0 (51)	36,0 (108)
Ne sais pas	12,7 (19)	6,7 (10)	9,7 (29)
**Attitudes favorables à la PFPPI**			
Empêche une nouvelle grossesse	71,6 (53)	87,6 (78)	79,6 (131)
Espacement des naissances	40,5 (30)	61,8 (55)	51,1 (85)
Epanouissement de la femme	20,3 (15)	40,4 (36)	51 (30,5)
Epanouissement sexuel du couple	21,6 (16)	38,2 (34)	29,9 (50)
Attitudes défavorables à la PFPPI			
Impact sur le lait maternel	54,4 (31)	76,5 (39)	65,4 (70)
Infertilité de la femme	19,3 (11)	33,3 (17)	26,3 (38)
Stérilisation de la femme	22,8 (13)	29,4 (15)	26,1 (28)
Abstinence en PPI	28,1 (16)	15,7 (8)	21,9 (24)
Préférence des méthodes traditionnelles	14,0 (8)	3,9 (2)	8,9 (10)
Interdiction religieuse/Culturelle	8,8 (5)	5,8 (3)	7,3 (8)
**Alternative future d’utilisation d’une MCM en PPI**			
Non	58,7 (88)	51,3 (77)	55,0 (165)
Oui	39,3 (59)	43,3 (65)	43,3 (124)
Utilise déjà	2,0 (3)	5,3 (8)	3,7 (11)
**Raisons de refus**			
Santé de la mère et de l’enfant	76,1 (67)	65,0 (50)	70,5 (117)
Abstinence post-partum	20,4 (18)	27,3 (21)	23,8 (39)
Désir d’un autre enfant	7,9 (7)	5,2 (4)	6,5 (11)
Situation économique	1,1 (1)	2,6 (2)	1,8 (3)
Opposition du partenaire		23,4 (18)	

**Figure 3:**
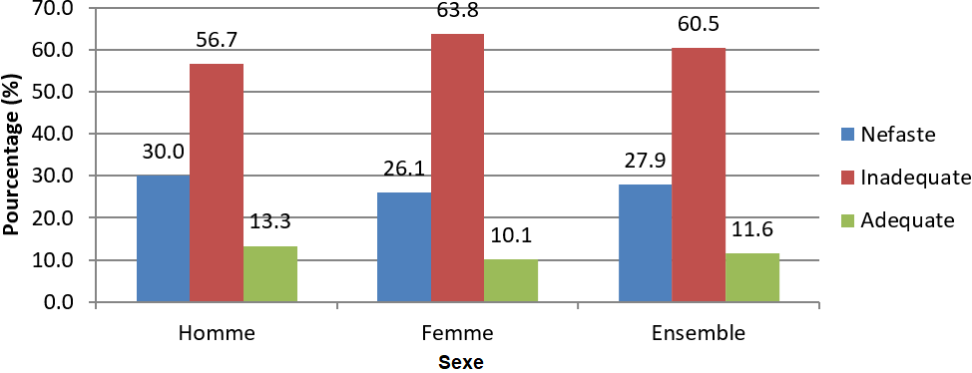
Pratiques contraceptives en PPI en fonction du sexe

**Pratiques contraceptives en PPI:** des 43,1% qui ont affirmé avoir des rapports sexuels en PPI, 57,4% pratiquaient une contraception moderne. L'unique méthode utilisée était le préservatif. Les sources d'information et d'approvisionnement variaient d'un individu à l'autre: 54% prenaient l'initiative seuls et 17,7% tenaient compte de l'avis d'un personnel de santé (Tableau 3). Une proportion non négligeable de 25,5% ne pratiquait aucune contraception. Très peu d'hommes (8,7%) étaient présents à la sortie de la maternité de leur conjointe. Les conseils reçus à la sortie de la maternité étaient d'ailleurs très peu relatifs à la PF (10,7%), le plus fréquent était le rendez-vous à la vaccination (80,2%). Les « autres » conseils (22%) avaient le plus trait à une proscription de rapports sexuels en PPI et à rendez-vous pour la PF, 06 semaines après l'accouchement (Tableau 3). L'analyse globale des pratiques selon la grille de Essi *et al.* [19] a montré que plus de la moitié des couples qui avait des rapports sexuels en PPI avait des pratiques contraceptives inadéquates (60,5%) (Figure 3).

**Tableau 3:** Pratiques sexuelles et contraceptives des couples en PPI

Variables	Hommes [% (n)]	Femmes [% (n)]	Total [% (n)]
**Reprise des rapports sexuels en PP**			
Un mois	27,3 (41)	38,0 (57)	32,7 (98)
03 mois	16,7 (25)	22,0 (33)	19,3 (58)
Arrêt de l'allaitement	24,0 (36)	12,0 (18)	18,0 (54)
Retour des couches	16,7 (25)	8,0 (12)	12,3 (37)
06 mois	2,7 (4)	12,0 (18)	7,3 (22)
Deux semaines après environ	7,3 (11)	6,0 (9)	6,7 (20)
Dès la sortie de la maternité	5,3 (8)	2,0 (3)	3,7 (11)
**Précautions en PPI (dès sortie maternité à un mois)**			
Méthodes modernes	50,0 (30)	64,6 (42)	57,3 (72)
Méthodes naturelles	18,3 (11)	13,8 (9)	16,0 (20)
Aucune	25,0 (15)	26,1 (18)	25,5 (33)
Méthodes traditionnelles	3 (5,0)	0 (0,0)	2,5 (3)
**Source d’information pour méthodes modernes**			
Initiative en couple	46,9 (30)	61,0 (58)	54,0 (88)
Personnel de santé	39,1 (25)	29,5 (28)	34,3 (53)
Entourage	14,0 (9)	9,5 (9)	11,7 (18)
Prise de décision			
Couple	70,0 (105)	68,0 (102)	69,0 (207)
Homme	23,3 (35)	10,0 (15)	16,6 (50)
Femme	6,0 (9)	22,0 (33)	14,0 (42)
Entourage	0,7 (1)	0,0 (0)	0,3 (1)
**Présence à la sortie de la maternité (hommes)/counseling sur PFPPI (femmes)**			
Jamais	77,3 (116)	55,3(83)	
Parfois	14,0 (21)	19,3 (29)	
Toujours	8,7 (13)	25,3 (38)	
**Conseils reçu à la sortie de la maternité**			
RDV à la vaccination	61,3 (19)	84,2 (123)	80,2 (142)
Type d'allaitement	48,4 (15)	21,9 (32)	26,6 (47)
Autres	19,4 (6)	22,6 (33)	22,0 (39)
Référence au service de PF	9,7 (3)	11,0 (16)	10,7 (19)

**Figure 4:**
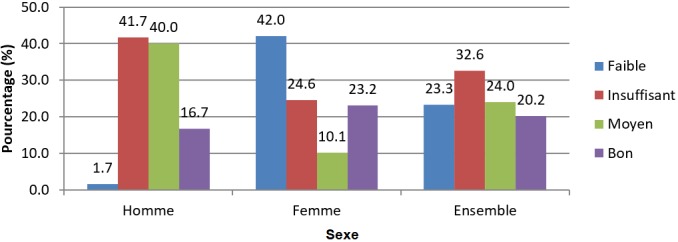
Compétences des couples en matière de PFPPI

**Compétences des couples en matière de PFPPI:** l'évaluation des compétences des enquêtés par association de leurs connaissances, attitudes et pratiques contraceptives selon la grille de Essi *et al.* [19] a montré que 32,6% avaient un niveau de compétence insuffisant; 24% un niveau moyen; 23,3% un niveau faible et 20,2% un bon niveau de compétences en matière de PFPPI. La représentativité par sexe variait d'un niveau à l'autre (Figure 4).

## Discussion

**Connaissances de la planification familiale en post-partum immédiat:** si l'on considère la connaissance de manière spontanée de l'utilisation des MCM en PPI, le niveau de connaissance est faible (37,6%), mais beaucoup moins que lorsqu'on demande aux répondants de spécifier la méthode, la source d'information, le moment de réception de l'information et la susceptibilité à la fécondabilité chez une femme en post-partum. La distinction entre connaissance spontanée et connaissance après spécification de toutes ces informations permet de se rendre compte du très faible niveau de bonnes connaissances en PFPPI par les personnes en couple dans le DS de Biyem-Assi (17%), avec une représentativité 2,6 fois inférieure chez les hommes; et du niveau élevé (56,7%) de mauvaises connaissances aussi bien chez les hommes que chez les femmes. La très faible présence des hommes à la sortie de la maternité de leur conjointe (8,7% seulement) pourrait être la cause de l'écart constaté sur le niveau des bonnes connaissances, car le counseling fait à la sortie de maternité peut parfois inclure des messages relatifs à la PF.

En effet, la principale méthode de contraception utilisable en PPI évoquée par les répondants était le préservatif (100%). Avec les multiples campagnes de lutte contre le VIH/Sida et les grossesses indésirées, le préservatif est devenu la méthode la plus connue dans la population. Etant donné qu'il ne nécessite pas l'avis d'un personnel de santé pour son utilisation, et que l'accessibilité autant financière que géographique ne pose pas de réel problème, la plupart des gens croient suffisamment savoir en matière de contraception. Le personnel de santé était la source d'information principale pour les femmes (75,4%). Les mêmes résultats ont été obtenus par Matungulu *et al.* [20] à Mumbunda en République Démocratique du Congo en 2015, où les formations sanitaires étaient à 75,6% la source principale de réception de l'information sur la PF par les femmes en union. Les hommes quant à eux se référaient beaucoup plus aux messages des média (37,5%), résultats identiques à ceux obtenus par Vouking *et al.* [21] au Nigéria en 2013, où 93% des hommes avaient comme principale source d'information la radio. Ceci pourrait également expliquer le meilleur niveau de bonnes connaissances chez les femmes. En effet, elles utilisent les services de santé pour des consultations prénatales (CPN), l'accouchement ou des consultations postnatales, augmentant ainsi leur chance de recevoir la bonne information sur la PFPPI. Mais au regard du moment de réception de l'information, l'on peut se rendre compte que beaucoup ne distinguent en réalité pas la PFPPI de la PFPP. Seuls ceux ayant reçu l'information immédiatement après la naissance de l'enfant peuvent être crédibles à cet effet. Ils ne représentent que 1,7% de l'échantillon.

Le retour des couches était l'indicateur premier sur lequel se basait la majorité de l'échantillon (46%) pour déterminer si une femme peut de nouveau concevoir après un accouchement. Les mêmes résultats ont été obtenus par des études coordonnées par Family Health International au Ghana, en Inde, au Rwanda et en Zambie [22]. Dans ces pays, la moitié des femmes en post-partum enquêtées ne savaient pas qu'elles pouvaient concevoir tant qu'elles étaient aménorrheïques, et attendaient le retour des couches pour initier la contraception. Il y a pourtant de nombreuses années qu'il a été démontré que le retour des couches n'est pas l'indicateur approprié pour déterminer la susceptibilité de fécondabilité chez une femme en PP. L'étude de Kennedy et Visness [23] dans 16 pays de l'Afrique Centrale, de l'Est, du Nord et de l'Amérique latine a montré que 6 à 11% des femmes en aménorrhée du PP ont contracté une grossesse. La persistance de ces croyances dans l'échantillon étudié pourrait se justifier par l'absence d'information des femmes/couples en suites des couches, et même penser que nombreux sont ceux qui ont un faible niveau de connaissance sur le cycle menstruel en général. Les résultats de cette étude montrent d'ailleurs que le counseling fait à la sortie de la maternité n'inclut presque pas les informations concernant le retour à la fécondité en PP.

**Attitudes vis-à-vis de la PFPPI:** l'opinion personnelle qu'à un individu de l'utilisation des MCM en période PPI est fortement influencée par les connaissances qu'il a de la PFPPI et par les rumeurs et croyances populaires. Bien que le niveau de connaissance de notre échantillon sur la PFPPI soit faible, plus de la moitié de l'échantillon (54,3%) avait tout de même conscience des avantages qu'elle peut avoir dans la vie familiale et sociale d'un couple. Des 36% des individus qui ne percevaient pas la période PPI comme moment approprié pour l'initiation d'une contraception, la plupart redoutaient leur impact sur le lait maternel (65,4%) et sur la santé de la mère (peur des effets secondaires, 26,1%). D'autres encore pensaient que les contraceptifs n'étaient pas nécessaires du fait d'une infécondité de la femme à cette période (26,3%). Ce qui pourrait s'expliquer par un manque d'information adéquate quant à la susceptibilité d'une grossesse dès le 25^ème^ jour PP, mais aussi à la possibilité d'utilisation de certaines méthodes dès la sortie de la maternité. Les mêmes résultats avaient été obtenus par Abera *et al.* [24] au Nord-Ouest de l'Ethiopie en 2015, où la non-perception du risque de grossesse en PP (49%) et l'absence du conjoint (16,8%) étaient les principales raisons pour lesquelles les femmes n'utilisaient pas de méthodes contraceptives. Ce qui témoigne également d'un manque d'éducation des populations en matière de santé sexuelle et reproductive.

Les prises de décision en ce qui concernait la PF en général se faisaient pour la plupart en couple (69%). C'est sur la base des mêmes résultats que Fassassi [25] démontre que l'interaction avec l'entourage, notamment entre conjoints constitue un facteur important de l'utilisation de la contraception à un moment donné. De même, Akoto *et al.* [26] ont montré que l'avis du conjoint sur la pratique contraceptive est la variable la plus influente dans deux pays africains: le Cameroun et le Kenya. Ainsi, le point de vue du conjoint est un élément déterminant pour l'approbation des MCM par certaines femmes. Dix-huit femmes, soit 23,4% de celles qui ne pourraient utiliser la contraception en PPI avaient pour principale raison l'opposition de leur conjoint. La prise en compte du partenaire masculin, ou mieux, du couple dans les programmes de PF est donc d'une importance cruciale.

L'analyse des attitudes globales de l'échantillon a montré que 38,3% avaient des attitudes approximatives vis-à-vis de la PFPPI. Ils pensaient en réalité que les rapports sexuels étaient possibles en PPI mais qu'une contraception n'était pas nécessaire ou aurait des inconvénients sur la santé. En effet, les barrières construites autour des MCM, l'influence négative de l'entourage et de certaines coutumes, la faible implication des hommes dans le volet de la planification familiale ont un impact certain sur les réticences vis-à-vis de ces méthodes. Des études ont montré que les femmes qui ont des attitudes favorables vis-à-vis des MCM ont plus de chance de les utiliser que celles ayant une attitude défavorable [20, 27, 28].

**Pratiques contraceptives des couples en PPI:** pour l'analyse des pratiques, n'ont été considérées que les personnes qui ont reconnu avoir des rapports sexuels en PPI, soit dès la sortie de la maternité à 06 semaines environ. Près de 44% ont reconnu reprendre avec les rapports sexuels aussitôt après un accouchement. Des résultats similaires ont été trouvés par Radziah *et al.* en Malaisie [29], Anzaku *et al.* au Nigéria [30] et Alum *et al.* en Uganda [31], où de nombreuses femmes avaient des rapports sexuels en PPI (avant 6 semaines après un accouchement). Etant donné que les couples ne vivent plus séparément après un accouchement comme par le passé, le tabou sexuel en PP disparait peu à peu et la reprise de rapports sexuels dans les semaines qui suivent un accouchement prend de plus en plus de l'ampleur. La faible représentativité des hommes (39,9%) par rapport aux femmes (46%) pourrait s'expliquer par le fait que les hommes préfèrent avoir recours à d'autres partenaires (27,6%) et que certaines femmes le font pour la satisfaction de leur conjoint (49,1%).

L'analyse des pratiques contraceptives selon la grille de Essi *et al.* [19] a montré une inadéquation de celles-ci dans ce groupe de personnes (60,5%), représentés majoritairement par les femmes (63,8% contre 56,7% chez les hommes). Globalement ces pratiques inadéquates pourraient s'expliquer par le manque d'information et de documentation sur la PFPPI au Cameroun. Le personnel de santé, notamment des Services Obstétriques, semble très peu informé à ce sujet étant donné que seuls 10,7% de notre échantillon a été référé à un service de PF à la sortie de la maternité. La plupart du personnel de santé demande aux couples de revenir 06 semaines après, pour la vaccination. Mais d'après les observations faites, même à ce moment, la PF n'est pas systématiquement abordée.

Seul 11,6% de l'échantillon a des pratiques contraceptives adéquates en PPI. En réalité, les seules méthodes utilisées par ces couples sont le préservatif et le retrait. Aucune autre méthode n'a été citée. Le préservatif est une méthode contraceptive fiable et adéquate en PPI mais il a l'inconvénient de nécessiter la coopération des deux partenaires et une disponibilité à chaque rapport sexuel. Ce qui n'est pas très évident pour des personnes vivant sous le même toit. Selon l'OMS et USAID [32], certaines femmes trouvent qu'il est difficile d'indiquer à leur partenaire, surtout lorsqu'ils vivent sous le même toit, qu'elles aimeraient utiliser un préservatif. Les hommes justifient généralement leur refus par une diminution de sensation lorsqu'ils utilisent le préservatif. Les résultats obtenus montrent que très peu de couples d'ailleurs (17,7%) prennent des conseils chez un personnel de santé. Plus de la moitié (54%) de ceux qui utilisent des méthodes modernes prennent seuls la décision et la plupart se ravitaillent dans les points de vente ordinaires. Le retrait quant à lui demande un réel engagement et une bonne communication au sein du couple. C'est une méthode contraceptive pas très efficace selon l'OMS bien qu'elle n'ait aucun effet secondaire, ni de risque pour la santé; car utilisé couramment, il peut survenir 27% de grossesses indésirées au cours de la première année [32]. Ce n'est donc plus une méthode très recommandée de nos jours.

**Compétences et besoin éducationnel des couples en matière de PFPPI:** les compétences en matière de PFPPI de l'échantillon étaient majoritairement insuffisantes (32,6%). Les mêmes résultats ont été obtenus dans le DS de Garoua, mais avec une proportion plus élevée de 70,4% [33]. Les compétences étaient meilleures chez les femmes (23,2%) que chez les hommes (16,7%). Le lieu par excellence d'acquisition de bonnes informations relatives à la PFPPI est la formation sanitaire. Mais les hommes sont non seulement très peu présents à la sortie de la maternité de leur conjointe, mais aussi, sont rarement ceux qui amènent l'enfant à la vaccination. Par conséquent, ils sont très peu exposés aux messages relatifs à la PF et sont moins susceptibles d'acquérir de bonnes connaissances pouvant améliorer leurs aptitudes en matière de PFPPI. Le besoin éducationnel en PFPPI est donc assez important, non seulement chez les hommes, mais également dans la population en général.

## Conclusion

Cette étude avait pour but de déterminer le niveau des compétences et le besoin éducationnel des couples en matière de PFPPI. Au regard des résultats obtenus (niveau des connaissances faible, attitudes approximatives et pratiques inadéquates), nous constatons qu'un grand effort reste à faire pour améliorer la pratique contraceptive dans le district de santé de Biyem-Assi. La période post-partum immédiate représente une fenêtre critique pour recevoir des services de PF, car elle a l'avantage d'une certitude que la femme n'est pas enceinte, qu'elle n'a pas encore de retour de couches et qu'elle peut être fortement motivée pour commencer une contraception efficace. Mais, un certain nombre de préjugés et de fausses idées persistent dans la population en ce qui concerne les MCM, et constituent un frein essentiel à la pratique contraceptive en général et en post-partum immédiat en particulier. Tout effort d'augmentation de la prévalence contraceptive, devrait donc cibler les facteurs tels que la peur des effets secondaires, la crainte d'une interaction entre les contraceptifs et le lait maternel, et les rumeurs sur l'irréversibilité des méthodes de contraception, afin d'optimiser l´atteinte de cet objectif. A cet effet, la formation du personnel de santé sur la PFPPI, la sensibilisation et l'éducation des couples pour l'amélioration de leurs pratiques contraceptives en post-partum immédiat s'avèrent nécessaires.

### Etat des connaissances actuelles sur le sujet

- La planification familiale est une intervention à haut impact et bas coût pouvant réduire la mortalité et la morbidité maternelle;
- Le choix d'une méthode contraceptive peut être fait immédiatement après un accouchement;
- Les femmes en post-partum ont le plus grand besoin non satisfait en planification familiale.

### Contribution de notre étude à la connaissance

- Bien que les méthodes contraceptives soient grandement connues dans le district de santé de Biyem-Assi, nombreux sont ceux qui ignorent que certaines de ces méthodes peuvent être utilisées immédiatement après un accouchement;
- Les couples dans le DS de Biyem-Assi ont encore des compétences très limitées en matière de PFPPI;
- Un moyen efficace d'atteindre un grand nombre de femmes/couples et ainsi de réduire le taux de besoins non satisfaits des couples en planification familiale du post-partum.

## Conflits d’intérêts

Les auteurs ne déclarent aucun conflit d'intérêts.
